# Characterization of a commercial plastic scintillator for electron FLASH dosimetry

**DOI:** 10.1002/acm2.14451

**Published:** 2024-07-01

**Authors:** Kyuhak Oh, Megan A. Hyun, Kyle J. Gallagher, Ying Yan, Sumin Zhou

**Affiliations:** ^1^ University of Nebraska Medical Center Omaha Nebraska USA

**Keywords:** dosimetry, electron ultra‐high dose rate (eFLASH), FLASH research extension (FLEX), plastic scintillator

## Abstract

**Purpose:**

This study investigated the potential of a commercially available plastic scintillator, the Exradin W2, as a real‐time dosimeter for ultra‐high‐dose‐rate (UHDR) electron beams. This work aimed to characterize this system's performance under UHDR conditions and addressed limitations inherent to other conventional dosimetry systems.

**Methods and materials:**

We assessed the W2's performance as a UHDR electron dosimeter using a 16 MeV UHDR electron beam from the FLASH research extension (FLEX) system. Additionally, the vendor provided a beta firmware upgrade to better handle the processing of the high signal generated in the UHDR environment. We evaluated the W2 regarding dose‐per‐pulse, pulse repetition rate, charge versus distance, and pulse linearity. Absorbed dose measurements were compared against those from a plane‐parallel ionization chamber, optically stimulated luminescent dosimeters and radiochromic film.

**Results:**

We observed that the 1 × 1 mm W2 scintillator with the MAX SD was more suitable for UHDR dosimetry compared to the 1 × 3 mm W2 scintillator, capable of matching film measurements within 2% accuracy for dose‐per‐pulse up to 3.6 Gy/pulse. The W2 accurately ascertained the inverse square relationship regarding charge versus virtual source distance with R^2^ of ∼1.00 for all channels. Pulse linearity was accurately measured with the W2, demonstrating a proportional response to the delivered pulse number. There was no discernible impact on the measured charge of the W2 when switching between the available repetition rates of the FLEX system (18–180 pulses/s), solidifying consistent beam output across pulse frequencies.

**Conclusions:**

This study tested a commercial plastic scintillator detector in a UHDR electron beam, paving the way for its potential use as a real‐time, patient‐specific dosimetry tool for future FLASH radiotherapy treatments. Further research is warranted to test and improve the signal processing of the W2 dosimetry system to accurately measure in UHDR environments using exceedingly high dose‐per‐pulse and pulse numbers.

## 1 INTRODUCTION

FLASH radiotherapy, delivering radiation at lightning‐fast speed (> 40 Gy/s), is a promising modality in treating cancer.^1^ The “FLASH effect” potentially improves the sparing of healthy organs and tissues for patients with cancer compared to conventional radiotherapy and decreases overall treatment time.^2^ Though the science is still evolving, FLASH has shown potential in lab experiments,^3^, ^4^ animal studies,^5^, ^6^ and even early human trials.^7^, ^8^

A significant roadblock between the promise and widespread use of FLASH radiotherapy is measuring how much radiation dose patients receive. Unlike conventional radiotherapy, existing dosimeters insufficiently address the ultra‐high dose rate (UHDR) environment produced by FLASH radiotherapy. While suitable for real‐time monitoring, ionization chambers lose accuracy at high absorbed dose and dose rates.^9^, ^10^, ^11^, ^12^, ^13^, ^14^ Radiochromic film and optically stimulated luminescent dosimeters (OSLDs) are excellent options for passive UHDR dosimetry but lack real‐time feedback and have maximum absorbed dose limitations (e.g., maximum dose of 15 Gy for OSLDs).^15^, ^16^ Yet to be established is the ideal, real‐time UHDR dosimeter.^17^, ^18^ In this study, we explored the potential of a real‐time, commercially available plastic scintillator for UHDR electron dosimetry using the novel FLASH research extension (FLEX) system.

## 2 MATERIALS AND METHODS

### 2.1 FLEX system

A commercial linear accelerator (Clinac 23EX, Varian Medical Systems, Palo Alto, CA, USA) was converted by the original vendor to the FLEX system, capable of delivering both conventional and UHDR electron beams. This unique system uses controlled pulse numbers (1–99 pulses) and pulse repetition frequencies (18–180 Hz) to generate a 16 MeV UHDR beam with approximately 180 Gy/s at the isocenter, reaching over 680 Gy/s at the gantry upper accessory mount.^19^, ^20^, ^21^

### 2.2 W2 and MAX SD system

This study investigated the promising potential of a commercial plastic scintillator (Exradin W2 Scintillator, Standard Imaging, Inc., Middleton, WI, USA) for real‐time UHDR electron dosimetry.^22^ The W2 is water‐equivalent and ideal for small‐field dosimetry.^22^ Paired with the MAX SD optical detection and signal processing unit, both the 1 × 1 mm and 1 × 3 mm W2 scintillators were evaluated in this study. The MAX SD was originally optimized for small signals, aligning with its intended use for small‐field dosimetry. However, UHDR radiation generates a large quantity of visible photons within the scintillator in a very short time. The MAX SD system uses photo‐detectors to convert the visible light signal into an electric signal for which the measured output of the electrometer is current or charge. Because the system was not originally designed to handle large currents, this leads to over‐ranging in the UHDR environment. To address this, the vendor provided a beta‐version firmware upgrade for the MAX SD, enhancing its ability to process the high signals produced by the UHDR environment and enabling it to handle currents up to 35 nA. Overall, the W2 dosimetry system features advanced processing capabilities, user‐friendly interfaces, online control, and easy data interpretation through Čerenkov‐corrected scintillation.^23^, ^24^

**FIGURE 1:**
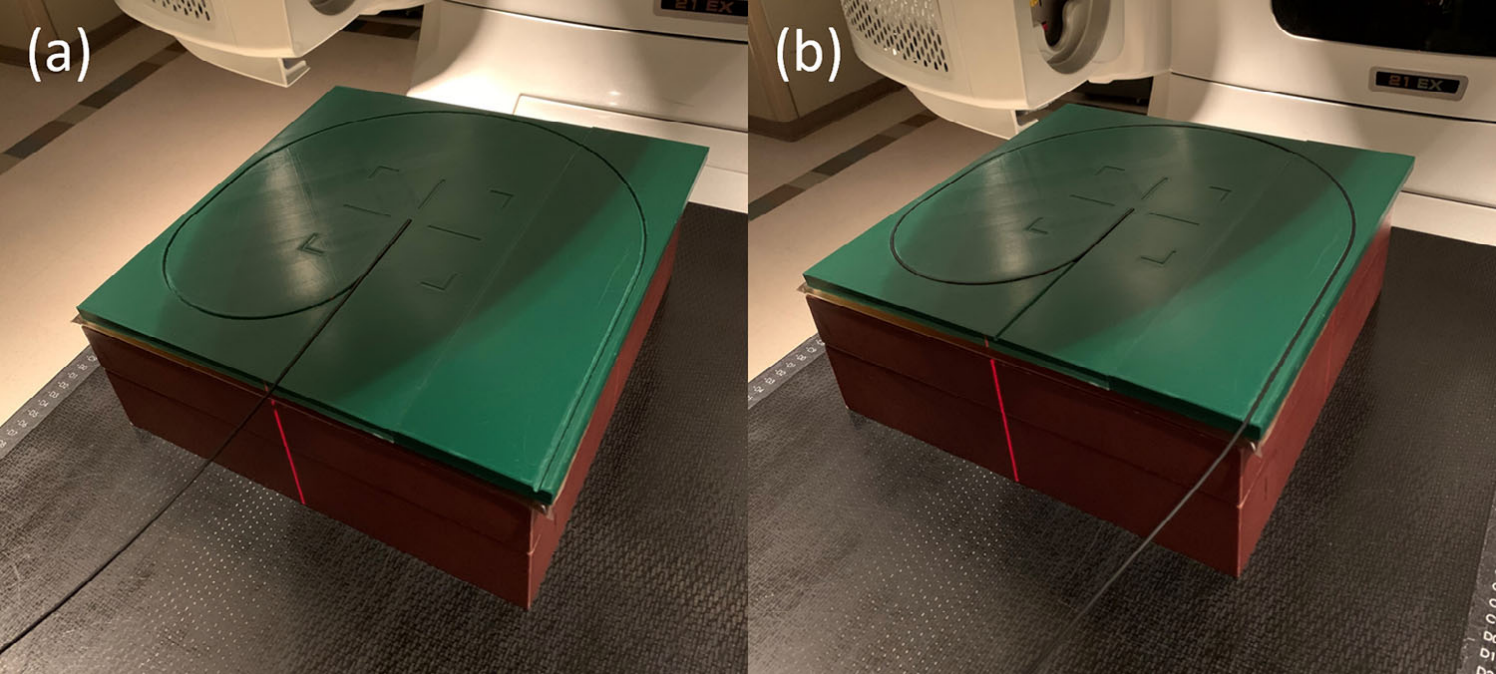
CLR measurement setups using the large‐field method for (a) minimized irradiation to the fiber and (b) maximized irradiation to the fiber. The plastic blocks with two different fiber pathways were 3D‐printed using polylactic acid filament (density of ∼1.2 g/cm^3^).

The MAX SD utilizes a chromatic spectral correction method to mitigate the Čerenkov light impact within the W2 detector system. This approach relies on the blue and green channel signal ratio, measured with both maximum and minimum fiber configurations as displayed in Figure 1.^21^ The following equation determines the Čerenkov light ratio (CLR):

$$CLR\;=\frac{Blue_{Max}-Blue_{Min}}{Green_{Max}-Green_{Min}}\;$$

where $Blue_{Max}$ ($Green_{Max}$) represents the blue (green) channel signal with the maximum irradiated fiber configuration and $Blue_{Min}$ ($Green_{Min}$) represents the blue (green) channel signal with the minimum irradiated fiber configuration (Figure 1).^22^, ^24^ Jacqmin et al.^22^ provide additional details on how the CLR is applied to correct for the Čerenkov light.

### 2.3 Dosimetric measurements

We deployed the W2 scintillator to analyze the beam properties of the FLEX system. Radiochromic film (GafChromic EBT‐XD, Ashland Inc., Wayne, NJ, USA) was used to validate the accuracy of the W2 system in the UHDR environment, particularly the detection efficiency of the W2 with increasing dose‐per‐pulse. Further tests used the W2 to characterize the 16 MeV FLEX system regarding:

- Source‐to‐dosimeter distance (SDD) dependence with virtual source correction
- Pulse linearity (i.e., output constancy with delivered number of pulses)
- Output constancy versus pulse repetition frequency (PRF)

#### 2.3.1 Detection efficiency

We tested the detection efficiency of the W2 plastic scintillators (1 × 1 mm and 1 × 3 mm) for a range of UHDR environments generated by the 16 MeV FLEX system. The gantry was positioned at 0 degrees (IEC scale) for all experiments with a field size of 10 × 10 cm^2^ without an applicator. To minimize air gaps, the W2 was positioned beneath 1‐cm of water‐equivalent bolus which is made from solid, homogeneous, and tissue‐equivalent gel (CQ Medical, Orange City, IA, USA). Additional water‐equivalent slabs were placed directly on top of the bolus to attain variable depths of measurement (3–9 cm). Furthermore, 10‐cm of water‐equivalent slabs were used downstream of the W2 for adequate backscatter conditions. The W2 system collected charge at an array of source‐to‐detector distances (61.6–132 cm) using a range of pulse numbers (1, 5, 10, and 43 pulses) at the highest PRF (180 Hz). Corresponding radiochromic film measurements of absolute dose enabled the conversion of the W2‐measured charge to dose. Furthermore, we compared measurements with the 1 × 1 mm W2 scintillator against a plane‐parallel ionization chamber (Advance Markus Electron Chamber, PTW, Freiburg, Germany)^13^, ^15^, ^25^ and OSLDs (NanoDot, Landauer Inc., Glenwood, Il, USA).

#### 2.3.2 Source‐to‐dosimeter distance (SDD) dependence

We explored the charge‐to‐distance relationship for both W2 scintillators positioned at 3 cm depth in the water‐equivalent slab phantom. We varied the SDD from 61.6 to 132 cm and delivered a single UHDR pulse at each distance.

#### 2.3.3 Pulse linearity

We tested the pulse linearity of the system by evaluating the collected charge of the 1 × 3 mm W2 scintillator with an increasing number of delivered pulses. The W2 was positioned at 136‐cm SDD and 6‐cm depth in the water‐equivalent slab phantom. We delivered 1, 2, 4, 6, 8, 10, 20, 30, 40, 50, 60, 70, 80, 90, and 99 pulses at 180 Hz PRF to assess the pulse linearity.

#### 2.3.4 Pulse repetition frequency effect

Similarly, we studied the effect of PRF on the system by positioning the 1 × 3 mm W2 scintillator at 136‐cm SDD and 6‐cm depth in the water‐equivalent slab phantom. We tested PRFs between 18 and 180 pulses/s (18, 36, 54, 72, 90, 108, and 180 Hz) by delivering 10 pulses at each PRF setting.

## 3 RESULTS

### 3.1 Detection efficiency

**FIGURE 2:**
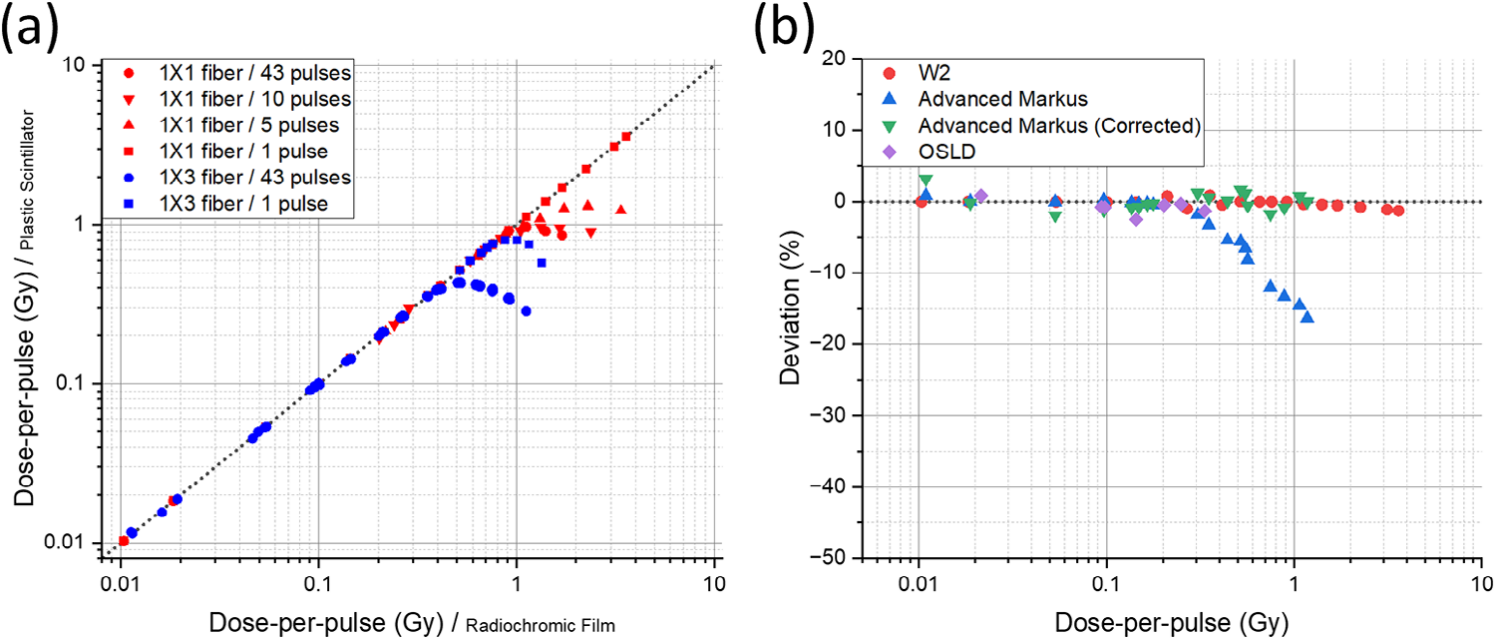
(a) Dose‐per‐pulse measured by both W2 scintillators with differing delivered number of pulses compared to radiochromic film; and (b) Percentage deviation of the measured dose‐per‐pulse by W2 1 × 1 mm scintillator, plane‐parallel ionization chamber, plane‐parallel ionization chamber (corrected) and OSLD from the reference dose‐per‐pulse determined by radiochromic film (single pulse delivery).

The signal from the larger, 1 × 3 mm W2 scintillator saturated at a lower number of delivered pulses and lower dose‐per‐pulse than the smaller, 1 × 1 mm W2 scintillator (Figure 2a). While the 1 × 3 mm W2 scintillator began to deviate from the linear relationship at 0.4 Gy/pulse (43 pulses) and 0.8 Gy/pulse (single pulse), the 1 × 1 mm W2 scintillator performed better, staying linear up to 0.9 Gy/pulse (5, 10, and 43 pulses) and 3.6 Gy/pulse (single pulse) (i.e., the maximum dose‐per‐pulse of the FLEX system). Furthermore, in comparing dose‐per‐pulse measurements across dosimetry systems (Figure 2b), the plane‐parallel ion chamber accurately matched the reference film up to 0.4 Gy/pulse (within 5%), while the 1 × 1 mm W2 scintillator remarkably demonstrated increased accuracy (within 2%) up to 3.6 Gy/pulse.

### 3.2 Source‐to‐dosimeter distance (SDD) dependence

**FIGURE 3:**
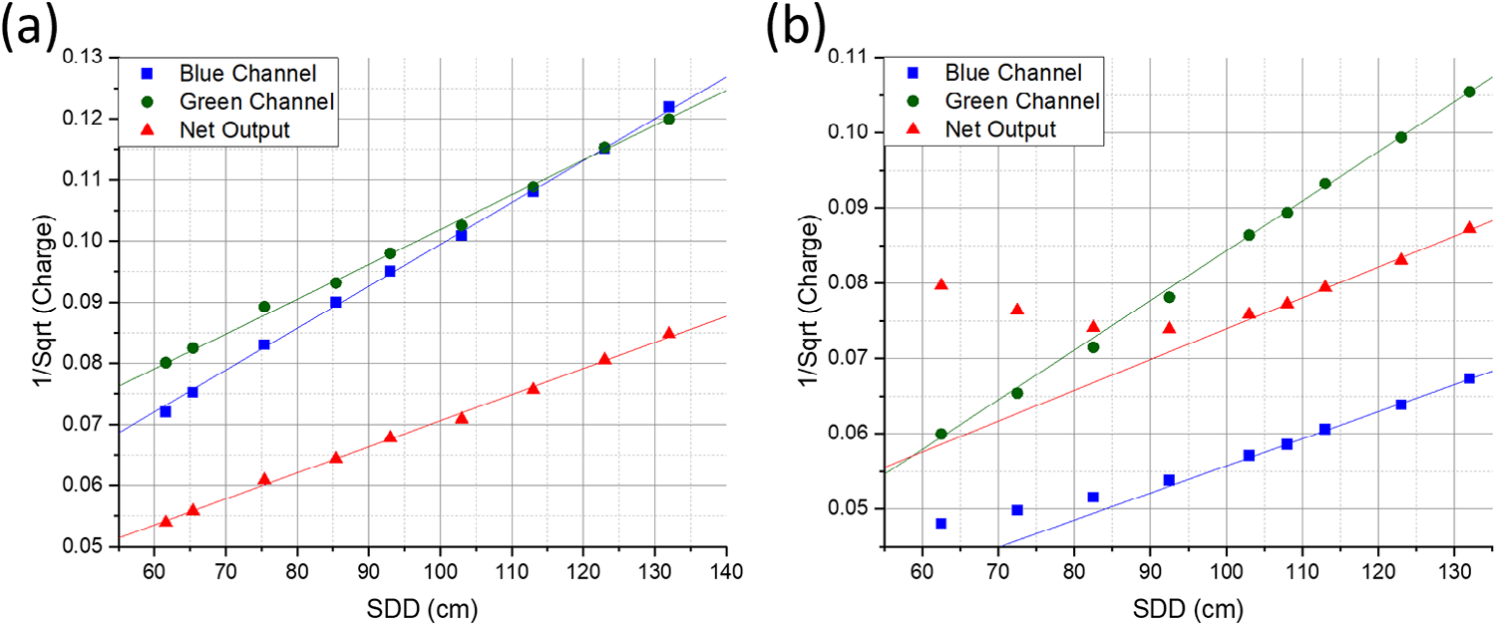
Source‐to‐dosimeter distance (SDD) dependence for the (a) 1 × 1 mm W2 scintillator and (b) 1 × 3 mm W2 scintillator. Shown is the comparison of the expected charge (continuous solid lines) calculated from the inverse square law accounting for the virtual source correction versus the actual measured charge by W2 plastic scintillators (discrete dots) at various SDDs.

First, we applied the virtual‐source distance correction to the FLEX machine's single pulse data using the 1 × 1 mm W2 scintillator (Figure 3a), and we observed the classical inverse square relationship between the collected charge and SDD. This strong relationship held for all three measurements (i.e., the blue channel, green channel, and Čerenkov‐corrected net output) with correlation coefficients (*R* ^*2*^) of 0.999, 0.998, and 0.998, respectively. Second, we analyzed the results of the 1 × 3 mm W2 scintillator for which the green channel continued to show this inverse square relationship. However, the blue channel and net output displayed this relationship only at larger SDDs (>92.5 cm for blue and >103 cm for net output). This discrepancy was due to the saturation of the 1 × 3 mm W2 scintillator at a higher dose‐per‐pulse (Figure 3b), because of the over‐ranging of the MAX SD system at higher currents.

### 3.3 Pulse linearity

**FIGURE 4:**
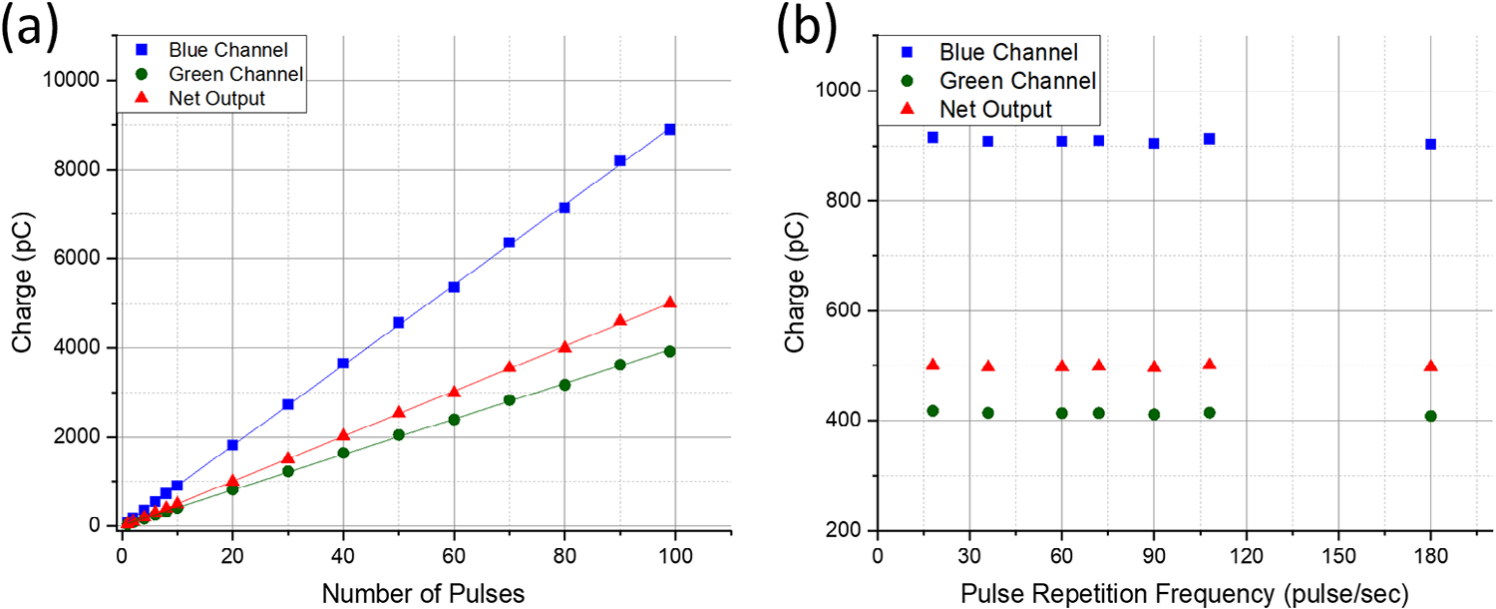
Displayed is the (a) pulse linearity test, i.e., measured charge versus number of delivered pulses, and (b) measured charge versus pulse repetition frequency (PRF) using the 1×3 mm W2 scintillator.

Using the 1 × 3 mm W2 scintillator, the collected charge for the blue channel, green channel, and net output all exhibited a proportional relationship with the number of delivered pulses, $R^{2}=\;1\;.000$ (Figure 4a).

### 3.4 Pulse repetition frequency effect

The plot in Figure 4(b) exhibits a horizontal trend line (i.e., zero slope) across all channels, indicating no significant change in the collected charge across all PRFs (18–180 Hz). This further demonstrates the stable output of the FLEX system at different pulse rates.

## 4 DISCUSSION

Our study highlighted a promising commercial plastic scintillator for real‐time UHDR electron dosimetry using the FLEX system. The W2 system responded exceptionally well to the UHDR beam characteristics, including pulse number, pulse repetition frequency, distance, and dose‐per‐pulse, particularly for the small W2 scintillator using low pulse settings. This implies that the 1 × 1 mm W2 scintillator is suitable for real‐time monitoring in UHDR applications using the FLEX system. Further research is warranted to test and improve the signal processing of the W2 system (i.e., the MAX SD) to accurately measure in UHDR environments using exceedingly high dose‐per‐pulse and pulse numbers.

In contrast to the W2, many conventional dosimetry systems fall short of accurate, real‐time monitoring of UHDR beams. Table 1 summarizes some advantages and disadvantages of using traditional dosimetry techniques, such as radiochromic film, TLD, OSLD, and ion chamber, in UHDR electron environments.

**TABLE 1:** Summary of characteristics for conventional dosimetry systems in UHDR electron environments.

Dosimetry system	Characteristics	References
Radiochromic film	Absorbed dose measurement Optimum dose range: 0.4–40 Gy Minimal dose rate dependence Energy dependency: <5% Post‐irradiation stabilization time needed Passive	6, 11, 25, 28
OSLD	Absorbed dose measurement Low directional dependence Dose rate independent up to ∼9000 Gy/s (average) and ∼150 kGy/s (instantaneous) Reusable Energy dependency: ∼5% Stable signal output after 8 min post‐irradiation Limited dose measurement up to 15 Gy Passive	28, 29
TLD	Absorbed dose measurement Dose rate independent up to ∼10^9^ Gy/s Energy dependent Reusable High sensitivity Post‐irradiation stabilization time needed Passive	11, 30, 31
Alanine	Absorbed dose measurement Wide dose range: mGy to kGy Dose rate independent up to ∼3 × 10^10^ Gy/s Reusable High sensitivity Post‐irradiation stabilization time needed Passive	6, 26, 31, 32
Ion chamber	Real‐time monitoring High sensitivity Easy to use Reproducible Stable Recombination effect for high dose rates (design dependent) Active	6, 9, 11, 25, 26, 28, 30

Researchers have also been approaching the challenging UHDR environment with innovative, real‐time dosimetry technologies:

- Ultra‐thin ionization chambers have demonstrated accuracy up to 5.4 Gy/pulse with minimal recombination losses (1.4%).^10^, ^33^
- Diamond detectors have also exhibited excellent accuracy up to 11.9 Gy/pulse with a deviation of less than 5%.^17^, ^34^
- Calorimeters and silicon carbide detectors hold promise for future real‐time monitoring.^35^, ^36^
- A dual beam‐current transformer offers a novel approach with a linear relationship to the dose rate.^37^

While the accuracy of many conventional scintillating dosimeters, such as phosphor point scintillators and some plastic scintillators, suffer at high dose rates,^38^ promising alternatives are emerging for accurate and real‐time UHDR beam monitoring.^18^ In our study, the original MAX SD firmware proved incompatible with ultra‐high dose rate dosimetry for both W2 scintillators. The resulting dose‐per‐pulse curve rapidly saturated and suffered even in the lower dose range. However, a beta‐version firmware upgrade provided by the vendor considerably improved the W2/MAX SD performance for UHDR beams and increased the current range upper limit from a maximum of 100 pA (standard setting) to 35 nA (beta‐version upgrade). This upgrade demonstrated optimal performance with the smaller W2 scintillator and a low pulse setting. However, the larger W2 scintillator or higher pulse numbers (up to 43 pulses) can still cause over‐ranging issues, resulting in saturation of the system. This issue could be further addressed through additional modifications to the MAX SD, featuring a more comprehensive current range to prevent overloading. Once resolved, this could solidify the W2 plastic scintillator as a viable UHDR dosimetry option.^39^, ^40^ Lastly, the W2 scintillator has a minor inherent limitation: its optical fiber suffers from radiation degradation of approximately 2% per kGy.^23^

## 5 CONCLUSIONS

We demonstrated the feasibility of using a commercial plastic scintillator with optimized configuration and updated firmware for real‐time UHDR electron dosimetry using the FLEX system. This development paves the way for real‐time, patient‐specific dosimetry verification during FLASH radiotherapy, alongside other potential applications.

## AUTHOR CONTRIBUTIONS

Kyuhak Oh, Megan A. Hyun, Kyle J. Gallagher, Ying Yan, and Sumin Zhou contributed to the conception and design of the project, as well as conducting all experiments, corresponding data analysis, and writing, reviewing, and editing the manuscript. Ying Yan and Sumin Zhou (Senior author) are the corresponding authors for this work.

## CONFLICT OF INTEREST STATEMENT

University of Nebraska Medical Center is a member of the Varian FlashForward Consortium.
